# Quercetin solubilisation in bile salts: A comparison with sodium dodecyl sulphate

**DOI:** 10.1016/j.foodchem.2016.05.034

**Published:** 2016-11-15

**Authors:** Maria Buchweitz, Paul A. Kroon, Gillian T. Rich, Peter J. Wilde

**Affiliations:** Institute of Food Research, Norwich Research Park, Norwich NR4 7UA, UK

**Keywords:** Quercetin’s pK_a1_, Micelles, UV–visible spectra, Pyrene fluorescence, Bioavailability

## Abstract

To understand the bioaccessibility of the flavonoid quercetin we studied its interaction with bile salt micelles. The environmental sensitivity of quercetin’s UV–visible absorption spectrum gave information about quercetin partitioning. Two quercetin absorption peaks gave complementary information: Peak A (240–280 nm) on the intermicellar phase and Peak B (340–440 nm) on the micellar phase. Thus, by altering pH, we showed that only non-ionised quercetin partitions into micelles. We validated our interpretation by studying quercetin’s interaction with SDS micelles. Pyrene fluorescence and the quercetin UV–visible spectra show that the adsorption site for pyrene and quercetin in bile salt micelles is more hydrophobic than that for SDS micelles. Also, both quercetin and pyrene reported a higher critical micelle concentration for bile salts than for SDS. Our method of using a flavonoid as an intrinsic probe, is generally applicable to other lipophilic bioactives, whenever they have observable environmental dependent properties.

## Highlights

• Quercetin partitions into bile salt and SDS micelles.
• Quercetin’s UV–visible spectra reflect its environment and micelle formation.
• Quercetin’s binding site is more hydrophobic in bile salt micelles.
• Pyrene fluorescence confirms quercetin’s spectral data.
• The results contribute to understanding quercetin’s low bioavailability.

## Graphical abstract

**Figure:**
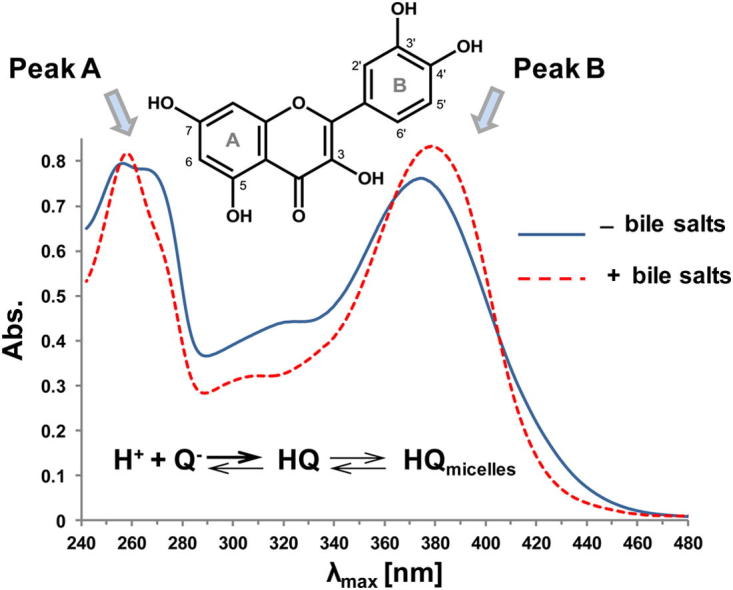

## 1 Introduction

The polyphenol quercetin is present in a large variety of fruits and vegetables in the form of quercetin glycosides. When it is consumed, enzymes in the small intestine convert these to the aglycone and this is absorbed by the enterocytes. Here quercetin undergoes transformations to conjugates and other derivatives, which have beneficial effects on target tissues (Guo & Bruno, 2015). Amongst the flavonoids, quercetin is one of the most potent antioxidants (Apak et al., 2004, Kim et al., 2006, Rice-Evans et al., 1996). Other therapeutic properties that have been attributed to quercetin include anticancer (Kobayashi, 2002, Sak, 2014), anti-inflammatory (García-Mediavilla et al., 2007, Hamalainen et al., 2007) effects and prevention of cardiovascular disease (Perez-Vizcaino, Duarte, & Andriantsitohaina, 2006). However, when consumed in either plant material or pure form, its bioavailability is low (Azuma, Ippoushi, Ito, Higashio, & Terao, 2002). This is understandable on the basis of its low aqueous solubility and it has been found that its absorption is enhanced by consuming it in a high fat meal (Guo et al., 2013, Lesser et al., 2004, Perez-Vizcaino et al., 2006). However, although it is described as a hydrophobic compound, amongst the flavonoids it has one of the lowest P values (octanol–water partition coefficient) (Rothwell, Day, & Morgan, 2005), implying its partition into hydrophobic phases is limited.

Solubilisation of hydrophobic molecules in the small intestine, so they become bioaccessible for absorption, is achieved by their partition into micelles. These are a mixture of simple and mixed micelles: the former consisting of bile salts and the latter of bile salts with lipids and their hydrolysis products (Hernell, Staggers, & Carey, 1990). To understand the basis of quercetin’s poor bioavailability, we have been studying its solubilisation in simple and mixed micelles relevant to the small intestine lumen.

In the course of our studies we found that the UV–visible absorption spectra of quercetin reflected its solubilisation and environment, and could therefore be used to monitor the process of micelle formation. In this paper we report on the interaction of quercetin with simple bile salt micelles under physiological conditions of pH, ionic strength and temperature. Due to the rigid ring structure of bile salts, their micelles are structured differently to those formed by conventional surfactants. The bile salt micelles can vary greatly in size, shape and aggregation number, and their formation is only weakly co-operative. They form dynamic structures with internal mobility, a variety of packing orientations and rapid exchange between the free and micelle-bound state (Dominguez et al., 2000, Madenci and Egelhaaf, 2010, Warren et al., 2006). Therefore, to be able to probe directly the interactions between micelles with a bioactive, such as quercetin by using the intrinsic properties of the bioactive itself offers great advantages. This approach will yield useful insights into the interactions between the bile salt micelles and the bioactive molecule without the aid of either added probe molecules, or physical methods.

For comparison and for validation of our approach, we also used a conventional surfactant, sodium dodecyl sulphate (SDS), which has been shown to solubilise quercetin (Liu & Guo, 2006). These workers’ studies were done under non-physiological conditions and were not designed to measure a critical micelle concentration (cmc). Our studies showed that under physiological conditions, just as for bile salts, quercetin’s UV–visible spectrum could report on the cmc of SDS.

**Fig. 1:**
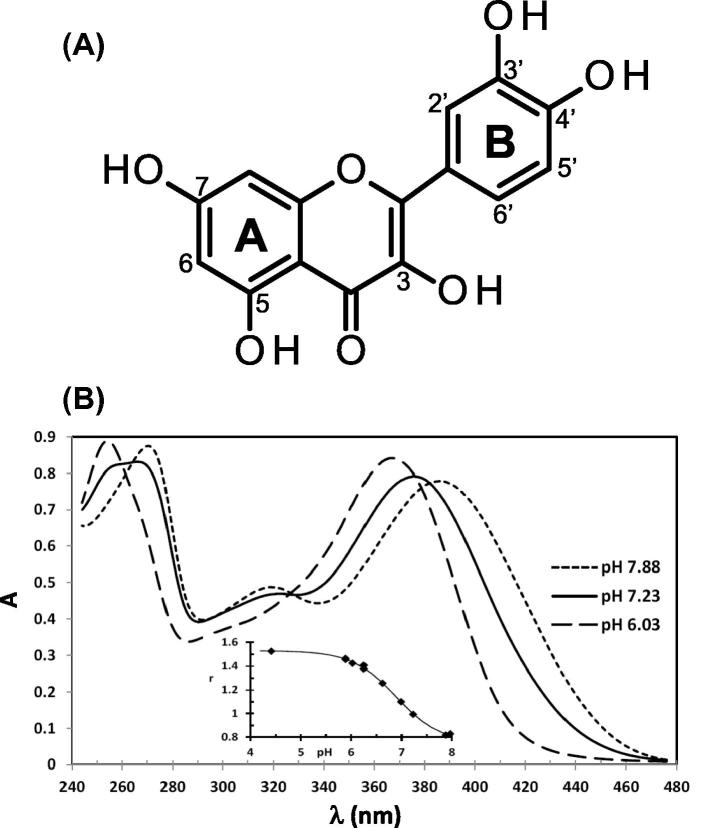
(A) The structure of Quercetin, and (B) the UV–visible absorption spectra of quercetin in DPBS at different pH’s at constant ionic strength. The pH values selected show the characteristic changes in spectra and in particular the peak at 270 nm due to Q^−^ at pH 7.88.The insert shows the data for one experiment for r = (A_max(254–257)_/A_270_) as a function of pH.

The structure of quercetin (3,5,7,3′,4′-pentahydroxyflavone) is shown in Fig.1A. It consists of two aromatic rings (A and B) linked by a γ-pyrone ring. The most acidic hydroxyl group is predicted to be that on the 7 position of the A ring (Musialik, Kuzmicz, Paawlowski, & Litwinienko, 2009). One would anticipate that quercetin binding to negatively charged micelles, such as those formed from bile salts and SDS, would depend on its ionisation state. Reported measurements of the pK_a1_ (the first dissociation step) in aqueous solutions cover a wide range from 3.3 to 8.2 (summarised by Mezzetti, Protti, Lapouge, & Cornard, 2011). However, none of these published pK_a1_ values were acquired under precisely the same solution conditions as we used in this present study. Therefore, to understand the interaction of quercetin with the micelles we needed an accurate measurement of pK_a1,_ which we obtained using the environmental effect of pH on quercetin’s absorption spectrum. To confirm and validate our conclusions we made further measurements of quercetin solubilisation in micelles under conditions more acidic than those pertaining to the duodenum, but relevant to the more distal small intestine. Pyrene fluorescence was used to verify the onset of micelle formation (cmc) and explore the polarity of the quercetin binding site in the micelles.

## 2 Materials and methods

### 2.1 Chemicals

Bile salts, sodium taurocholate (NaTC) and sodium glycodeoxycholate (NaGDOC), dimethyl sulphoxide (DMSO), dimethylformamide (DMF), propylene carbonate, glycerol and ethylene glycol were obtained from Sigma Aldrich (Gillingham, UK). DMSO was stored under argon. SDS was specially pure grade from BDH (VWR, Poole, UK). Dulbecco’s phosphate buffered saline (DPBS), Ca^2+^ and Mg^2+^ free, (10×) was from Gibco, Life Technologies Corp (Paisley, UK). Other reagents were of analytical or HPLC grade.

### 2.2 Preparation of solutions

Quercetin, as an antioxidant, is inevitably prone to oxidation, including photo-oxidation (Takahama, 1985). For reproducible results it was necessary to remove oxygen from solutions and restrict their exposure to light. Solutions were de-oxygenated by sonication and kept in amber glassware pre-filled with argon. Quercetin, at 1000 times the required final concentration, was dissolved in DMSO and diluted into DPBS ± surfactants, so the DMSO concentration was 0.1%. The ionic strength of DPBS derives primarily from NaCl (0.14 M) and Na_2_HPO_4_ (8.1 mM). The solvents used for the solvent study were dried over 4 Å (DMF and DMSO) and 3 Å (propylene carbonate) molecular sieves. Glycerol was dried over Na_2_SO_4_. The ethylene glycol was anhydrous. All measurements were made at 37 ± 0.5 °C.

### 2.3 Measurement of UV–visible absorption spectra

Spectra were measured using 1 cm quartz cells in an Uvicon xs spectrophotometer (NorthStar Scientific, Potton, UK) from 240 to 485 nm. The resolution was 0.1 or 0.25 nm with a scan speed of 50 or 100 nm/minute, respectively. Data were processed with a medium smooth using the instrument’s software. Spectra were measured against a reference of 0.1% DMSO in DPBS.

### 2.4 Determination of pKa1

In the UV–visible range of 240–500 nm, quercetin has two main absorption bands: band A (240–280 nm) and band B (340–440 nm). The spectra are pH dependent reflecting its ionisation state. Ionisation constants are ionic strength dependent. The ionic strength of DPBS at pH 7.15 is calculated to be 159.1 mM, with the phosphate salts contributing 18.5 mM. Using the published data (Dawson, 1969) for phosphoric acid buffers of constant ionic strength, spectra were measured for 45 μM quercetin dissolved in a series of solutions of different pH’s, containing an ionic strength of 18.5 mM KH_2_PO_4_ plus Na_2_HPO_4_ made up to 159.1 mM with NaCl.

As described in the results section there was evidence that the first dissociation of quercetin occurs under physiological conditions. We write the dissociation constant as:

(1) $${\text{K}}_{\text{a}1}=\frac{\left[{\text{H}}^{+}]\times [{\text{Q}}^{-}\right]}{\left[\text{HQ}\right]}=\frac{\left[{\text{H}}^{+}]\times [{\text{Q}}^{-}\right]}{\left({\text{Q}}_{\text{TOT}}-[{\text{Q}}^{-}]\right)}$$

where [ ] indicate concentrations and the total quercetin concentration Q_TOT_ is the sum of non-ionised quercetin (HQ) and its ionised form (Q^−^).

The fractions of HQ and Q^−^ are respectively:

(2a) $${\text{f}}_{\text{HQ}}=\frac{\left[{\text{H}}^{+}\right]}{\left([{\text{H}}^{+}]+{\text{K}}_{\text{a}}\right)}$$

and

(2b) $${\text{f}}_{{\text{Q}}^{-}}=\frac{{\text{K}}_{\text{a}}}{\left([{\text{H}}^{+}]+{\text{K}}_{\text{a}}\right)}$$

A method to determine K_a1_ from the UV–visible absorption spectra is to choose two wavelengths, λ_1_ and λ_2_, and use the ratio (r) of the two absorbances to fit the experimental data:

(3) $$\text{r}=\frac{{\text{A}}_{\lambda 1}}{{\text{A}}_{\lambda 2}}$$

(4) $$\text{r}=\frac{\varepsilon_{{\text{HQ}}_{\lambda 1}}\times [\text{HQ}]+\varepsilon_{{\text{Q}}_{\lambda 1}^{-}}\times [{\text{Q}}^{-}]}{{\text{ε}}_{{\text{HQ}}_{\lambda 2}}\times [\text{HQ}]+\varepsilon_{{\text{Q}}_{\lambda 2}^{-}}\times [{\text{Q}}^{-}]}$$

where $\varepsilon_{x\lambda i}$ are extinction coefficients.

Expressing the concentrations as f_HQ_ x Q_TOT_ and ${\text{f}}_{{\text{Q}}^{-}}\times {\text{Q}}_{\text{TOT}}$:

(5) $$\text{r}=\frac{[{\text{H}}^{+}]+{\text{c}}_{1}\times {\text{K}}_{\text{a}}}{{\text{c}}_{2}\times [{\text{H}}^{+}]+{\text{c}}_{3}\times {\text{K}}_{\text{a}}}$$

$$\text{where}\;c_{1}=\frac{\varepsilon_{{\text{Q}}_{\lambda 1}^{-}}}{\varepsilon_{{\text{HQ}}_{\lambda 1}}}\text{,}\;{\text{c}}_{2}=\frac{\varepsilon_{{\text{HQ}}_{\lambda 2}}}{\varepsilon_{{\text{HQ}}_{\lambda 1}}}\text{,}\;{\text{c}}_{3}=\frac{\varepsilon_{{\text{Q}}_{\lambda 2}^{-}}}{\varepsilon_{{\text{HQ}}_{\lambda 1}}}$$

We found peak A to be most sensitive to pH changes and λ_1_ and λ_2_ were chosen as 254–257 nm (the wavelength of maximum absorbance for HQ) and 270 nm (the wavelength of maximum absorbance for Q^−^), respectively, to fit the data.

### 2.5 Preparation of micelles

Micelles were made from either a mixture of NaTC and NaGDOC or SDS in DPBS. The pH of DPBS was adjusted to the required pH (7.15, 6.1 or 5.0) by the addition of 0.1 to 1 M HCl. NaTC is hygroscopic, so it was dried to constant weight and stored over calcium oxide. It was weighed into an argon-filled volumetric flask to which was added a weighed amount of NaGDOC so the NaGDOC/NaTC molar ratio was 1.12. This composition was selected to mimic the average hydrophobicity of bile salts in human bile, which was calculated from the composition of human bile (Staggers, Hernell, Stafford, & Carey, 1990) and the relative hydrophobicity of individual bile salts (Armstrong & Carey, 1982). DPBS at the relevant pH was added to give a concentrated stock solution (100–130 mM) and where necessary the pH was readjusted. Stock SDS solutions were made similarly at a concentration of 80 mM. The surfactant solutions were diluted into DPBS to which quercetin or pyrene were added at the required concentrations (see results section) and after another sonication, equilibrated at 37 °C.

### 2.6 Pyrene fluorescence to probe micelles

Pyrene partitions into micelles and as it moves from a polar aqueous phase into a more hydrophobic micelle its fluorescent properties change. At low pyrene concentrations, a convenient measurement of polarity is the ratio, F*_R_*, of the intensity of two of the fine structure peaks (III and I, around 383 and 372.5 nm respectively) in the emission spectrum (*Kalyanasundaram & Thomas, 1977*). Aliquots of pyrene in ethanol were evaporated to dryness under nitrogen and dissolved in micellar solutions with and without quercetin. With an excitation wavelength of 310 nm, emission spectra were measured on a Perkin Elmer LS55 Luminescence Spectrometer with excitation and emission slit widths of 2.5 nm. The pyrene concentration was 0.2 to 1.0 *μ*M, the higher concentrations being used in the presence of quercetin, which quenches the pyrene fluorescence.

### 2.7 Quercetin UV–visible spectra to probe micelles

Quercetin in DMSO was added with stirring to surfactant solutions (0 to 15 mM) to give final quercetin concentrations of 45 or 22.5 μM. After equilibration, UV–visible spectra were measured as described in Section 2.3.

### 2.8 Analysis of fluorescence and UV–visible spectra to determine cmcs

The optical properties of probes that reflect micelle formation as they partition into micelles typically show a sigmoid curve as the surfactant concentration, [S], increases. The sigmoid relationship between the properties of the probe and their concentration is conveniently described by a logistic equation, that describes the co-operative nature of surfactant aggregation to form micelles and the competitive effect as the micelles compete for pyrene, whose affinity for the micelles will depend on the conditions. This equation can be written as:

(6) $$\text{P}={\text{P}}_{0}+\frac{\left(\text{K}\times \Delta {\text{P}}_{\text{max}}\right)}{\left(\text{K}+{\text{e}}^{\left(-[\text{S}]\times \text{c}\times \Delta {\text{P}}_{\text{max}}\right)}\right)}$$

where P is the optical property (e.g. F_R_, in the case of pyrene fluorescence). P_0_ is its value in the absence of surfactant and ΔP_max_ is the maximum change in P. [S] is the surfactant concentration, K and c are constants.

The centre of the sigmoid curve is a point of inflection where:

$$\frac{{\text{d}}^{2}\text{P}}{\text{d}{\left[\text{S}\right]}^{2}}=0$$

At this midpoint we call the surfactant concentration [S]_1/2_:

(7) $${\left[\text{S}\right]}_{1/2}=\frac{-\text{lnK}}{\text{c}\times \Delta {\text{P}}_{\text{max}}}$$

The maximum slope is at [S]_1/2_:

(8) $${\left|\frac{\text{dP}}{\text{d}[\text{S}]}\right|}_{\text{max}}=\frac{\text{c}\times \Delta {\text{P}}^{2}}{2}$$

This equation was originally used to describe bile salt micelle formation associated with the solubilisation of carotenoids (Rich, Faulks, Wickham, & Fillery-Travis, 2003). It applies when P reflects an interaction of a probe molecule with micelle formation. As shown in the results section, just as for pyrene, Eq. (6) also describes features of quercetin’s UV–visible spectra as micelles form. For example, P can be a wavelength of maximum absorption of quercetin (λ_max_) or a ratio of absorbances at two different wavelengths (r = A_max(254–257)_/A_270_).

Different quantitative terms have been used to define the cmc: 1. the concentration at which surfactants start to aggregate (Carey & Small, 1969), 2. [BS]_1/2_ (Aguiar, Carpena, Molinar-Bolivar, & Ruiz, 2003), and 3. the concentration where P values reach a plateau (Madenci & Egelhaaf, 2010). Using probes to assess aggregation, criteria 2. and 3. have a dependence on the affinity of the probe for the micelles. Therefore, following Carey & Small we have called the cmc, the concentration at the onset of aggregation as reported by the probes. This leads to uncertainty for quercetin, which interacts with monomeric surfactants. We also report [BS]_1/2_, which gives discrete values and is dependent on both surfactant aggregation and affinity of the probe for the micelles.

## 3 Results

### 3.1 Impact of pH on UV–visible spectra of quercetin and determination of its pKa1

Fig. 1B shows the UV–visible spectra of 45 μM quercetin as a function of pH under physiological relevant temperature and ionic strength conditions (see Section 2.4). The most notable feature, as the pH increases, is the emergence of a shoulder at around 270 nm in band A becoming a peak at the highest pH. A plot of the absorbance ratio r of the two peaks in band A (A_max(254–257)_/A_270_) is shown in the inset to Fig.1B. As the pH is lowered quercetin becomes less soluble so we deduced the peak at 270 nm is due to quercetin’s first ionisation to form Q^−^. In addition as the pH is increased the wavelength of maximum absorption, λ_max_, for band B shifts to longer wavelengths and the band broadens.

The data from three independent experiments were fitted to Eq. (5) giving a pK_a1_ value of 7.08 ± 0.04 (mean ± SD). The associated constants c_1_, c_2_ and c_3_ were 0.828 ± 0.018, 0.654 ± 0.004 and 1.073 ± 0.003 respectively. Herrero-Martinez, Sanmartin, Rosés, Bosch, and Ràfols, (2005) measured quercetin’s pK_a1_ at 25 °C in aqueous solutions of 0.05 M ionic strength by capillary zone electrophoresis. Adjusting their value to our conditions of temperature and ionic strength using the Debye-Hückel equation (Bates, 1973) we get a value of 7.06 ± 0.12, which is in agreement with our measurements. The shift of λ_max_ for peak B is also consistent with a pK_a1_ around 7.1 (results are provided in the supplementary materials, Fig. S1A and B). Therefore, further experiments were performed at a physiological pH typical of the duodenum (pH 7.15), which is just above the pK_a1_, where 50% of the quercetin is in the Q^−^ form. Other pH values were chosen below the pK_a1_, at pH6.1 where nearly all (>95%) of the quercetin is in the HQ form, and at pH 5 where all the quercetin will be in the HQ form, and should have a greater affinity for micelles.

### 3.2 Pyrene fluorescence: cmc determination and micelle polarity

**Fig. 2:**
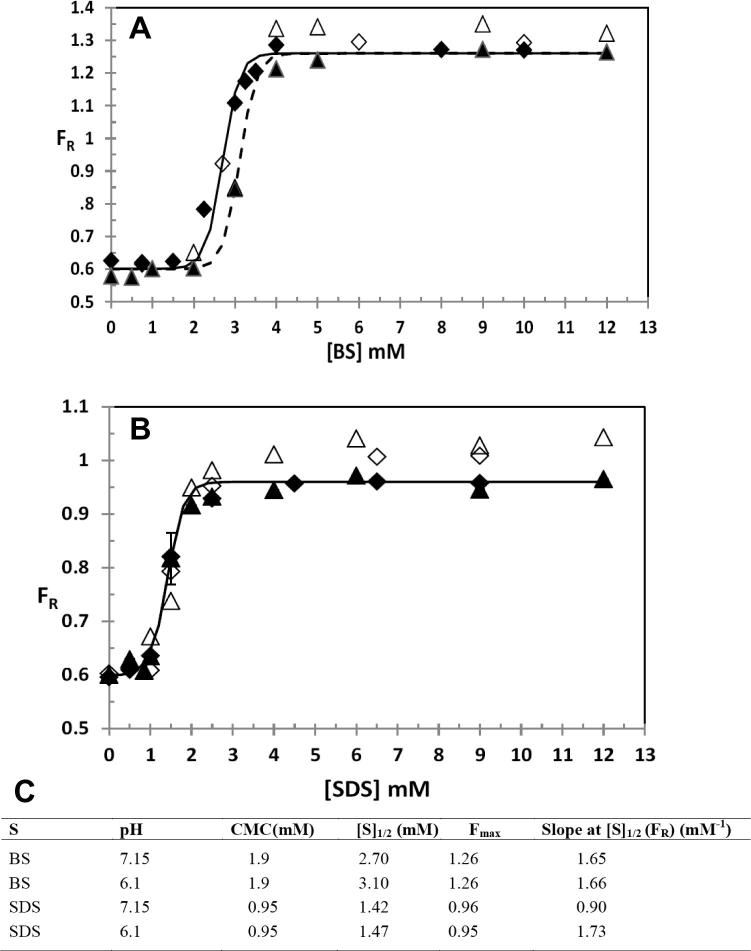
Change in the pyrene fluorescence intensity ratio, F_R_, for third and first vibronic peaks (F_III_/F_I_) as a function of bile salt (A) and SDS (B) concentration at different pH’s with and without quercetin (45 μM). ♦ pH 7.15 without quercetin, ♢ pH 7.15 with quercetin, ▴ pH 6.1 without quercetin, Δ pH 6.1 with quercetin. Solid line is for pH 7.15, dotted line for pH 6.1. Summary of analysis of pyrene fluorescence data (C), CMC, starting concentration of micelle formation; [S]_1/2_, inflection/ midpoint of sigmoid curve for micelle formation; F_max_, the maximum ratio of the intensity of the fine structure peaks at 383 and 372.5 nm; Slope at [S]_1/2_, fitting parameter for calculation of [S]_1/2_; results are for 2–3 experiments with SD for the values within 5% (cmc’s, slope, and [S]1/2) and 2% (F_max_).

Pyrene fluorescence measurements of BS and SDS solutions were unaffected by the presence of 0.1% DMSO. Further, the results were independent of pH (Fig. 2). Although it was found that the cmc and [BS]_1/2_ reported by pyrene is unchanged by the presence of quercetin, at higher BS and SDS concentrations, in the presence of quercetin, F_R_ values increase slightly. The effect is larger for SDS and for BS’s is only significant at pH 6.1.

### 3.3 Impact of BS and SDS on quercetin UV–visible spectra; determination of cmcs

**Fig. 3:**
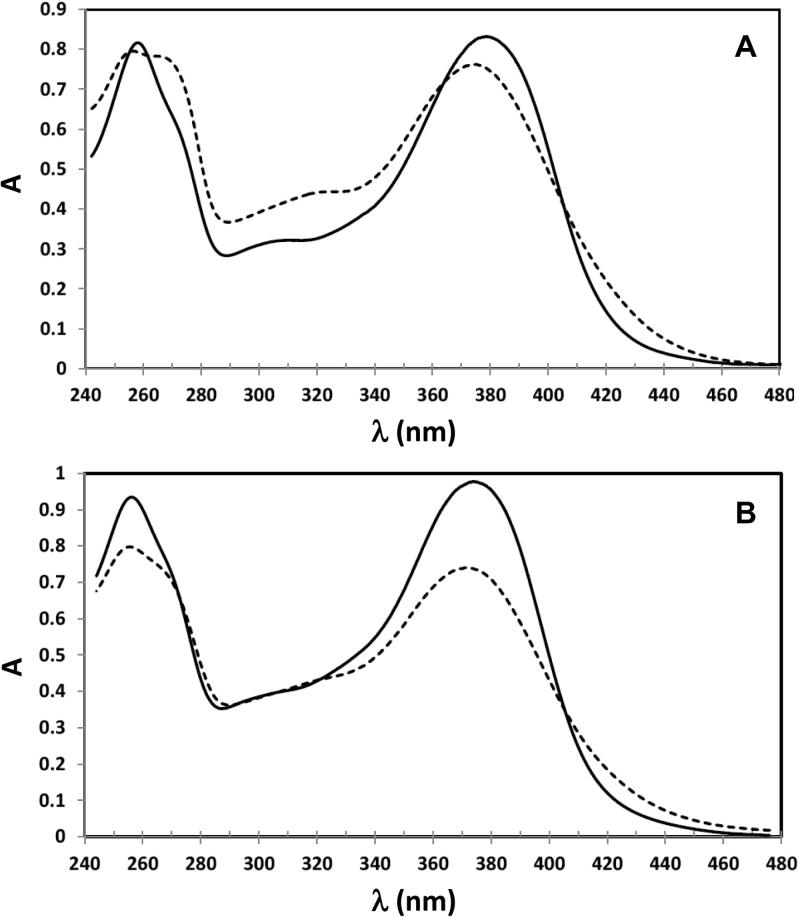
UV–visible absorption spectra of quercetin in DPBS at pH 7.15 without (dashed line) and with (solid line) addition of 12 mM BS (A) or SDS (B).

**Fig. 4:**
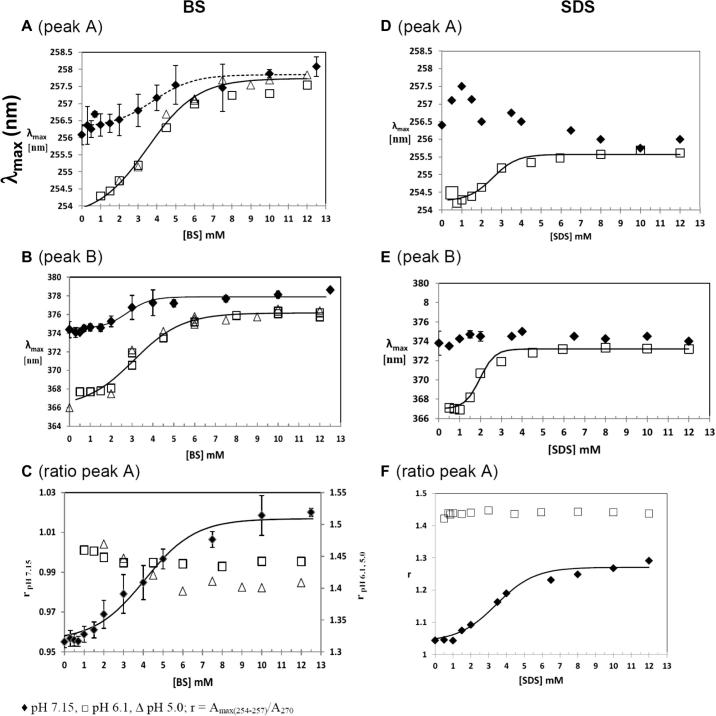
The effect of increasing BS (A–C) and SDS (D–F) concentration on the wavelength of maximum absorption (λ_max_) of quercetin for peak A (A, D) and peak B (B, E) and the change in the ratio r (C, F) of peak A at different pH values. ♦ pH 7.15, □ pH 6.1, Δ pH 5.0; r = A_max(254–257)_/A_270_

Fig. 3 shows UV–visible absorption spectra of 45 μM quercetin in the presence and absence of surfactants in DPBS (pH 7.15) at 37 °C. As the surfactant concentration increases the absorption of peaks A and B increases. For bile salts both peaks’ absorption maxima shift to longer wavelengths (Fig. 4A and B). Also, the shoulder around 270 nm of peak A decreases. The decrease in peak A’s shoulder is shown in Fig. 4C as the ratio r of the absorbance at its maximum to the 270 nm absorbance (A_max(254–257)_/A_270_). The increase of λ_max_ for both peaks show sigmoid curves, typical of cooperative micelle formation with similar BS concentrations at the onset of micelle formation. However, the slope is larger for the influence of increasing micelle formation on peak B compared to peak A.

As described in Section 3.1, a study of quercetin absorbance as a function of pH, showed that the absorbance at 270 nm was due to the ionisation of quercetin. The spectra of quercetin in bile salts at pH 7.15 were deconvoluted to show peak A consisted of two peaks with maxima at 270 and around 257 nm. For the same ΔP and surfactant, the maximum slope gives an indication of the partition of the probe into the surfactant micelles. The continuous lines in Fig. 4A–C show the fit of Eq. (6) to the data and the [BS]_1/2_ and cmc values are listed in Table 1. The latter are estimated from the surfactant concentration where the sigmoid curves indicate the start of fall in monomer concentration due to pre-micelle/micelle formation. The significance of [S]_1/2_ for band A being greater than [S]_1/2_ for band B is considered in the Discussion section together with the uncertainty of the cmc values.

Consistent with a pK_a1_ of 7.08, lowering the pH makes quercetin increasingly insoluble, particularly in the absence of surfactants. Therefore, working with 45 μM quercetin at pH 6.1 is only possible if surfactants are present. Halving the concentration to 22.5 μM quercetin had no effect on the λ_max_ values. At pH 5.0, to overcome quercetin’s limited solubility, we used 22.5 μM quercetin. (At this pH the ionic strength due to phosphate decreases by 5.5% compared to that at pH 7.15.) The effects of lowering the pH on quercetin’s spectra are shown in Fig. 4A–C. λ_max_ values increase more with bile salt concentration at pH 5.0 and 6.1 compared to pH 7.15

There is no significant difference between these data at the two lower pH’s where the percentage of Q^−^ are 0.0082% and 9.4% (calculated according to Henderson-Hasselbach equation), respectively. In addition, at lower pH’s, the enhancement of r for peak A disappears (Fig. 4C, right hand y axes) and peak B broadening with bile salt concentration is also not apparent (spectra not shown).

Fig. 4 shows that there are clear differences between BS and SDS and how they interact with quercetin. The bathochromic shifts in band A and band B at 6.1 are greater for BS (Fig. 4A and B) than for SDS (Fig. 4D and E). At pH 7.15, the shift is reduced for BS and completely disappears for SDS. For the peak ratio *r*, the trends are quite similar between BS (Fig. 4C) and SDS (Fig. 4F). However, at pH 7.15 the change in *r* is much greater for SDS at pH 7.15 than it is for BS. The sigmoidal increase in *r* disappears for both SDS and BS at the lower pH values. The differences between the behaviour of quercetin between BS and SDS suggest that the environments sensed by quercetin in the two types of micelles are different. This will be discussed in more detail in Section 4.1.

### 3.4 Solvent Studies: the relationship between λ_max_ for peak B and solvent polarity

**Fig. 5:**
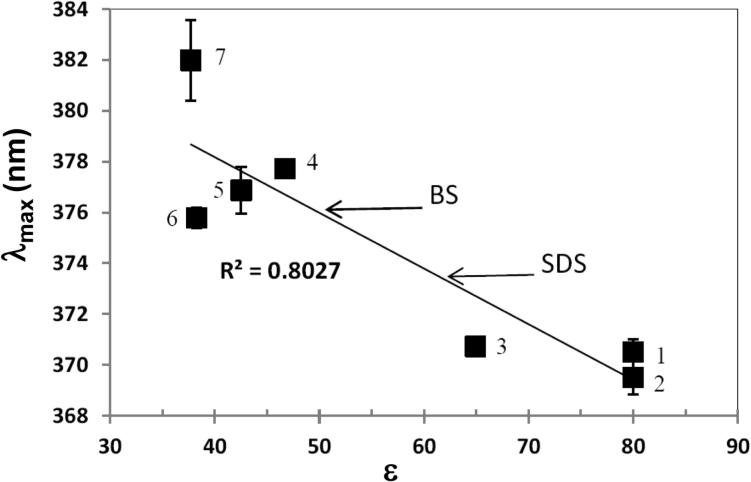
λ_max_ of peak B as a function of the dielectric constant ε for solvents of high polarity. 1, water; 2, water + 0.1% DMSO; 3, propylene carbonate; 4, DMSO; 5, glycerol; 6, dimethyl formamide; 7, ethylene glycol

To relate the wavelength shifts in the quercetin spectra to the polarity of the micellar environments we studied the spectra of quercetin in solvents of different dielectric constants. Fig. 5 shows the increase in the bathochromic shift of the maxima in peak B with decreasing solvent polarity. A decrease in F_R_ is associated with increased polarity (dipole moment) of the molecules surrounding pyrene (Kalyanasundaram & Thomas, 1977). Therefore, an increasing F_R_ ratio in the pyrene fluorescence spectra as well as bathochromic shift for peak B in the UV–visible spectra of quercetin indicate a more non-polar/ hydrophobic environment. The highest plateau λ_max_ values for quercetin in BS and SDS micelles at pH 5 and 6.1 are arrowed in Fig. 5. At the lower pH’s there is negligible contribution of Q^−^ to the spectra.

## 4 Discussion

### 4.1 General

Changes in quercetin’s UV–visible spectral properties as it partitions into SDS micelles have been studied before (Liu & Guo, 2006). Their work was done over a wider surfactant concentration range without using the data to measure a cmc. Also, we assume the measurements were made in water equilibrated with the atmosphere, where the pH would be so low that there would be no significant absorption due to Q^−^. An interaction of flavonoids with monomeric SDS has been previously reported (Naseem, Sabri, Hasan, & Shah, 2004). The interaction was postulated to be via H-bonding. Similarly, in this study we present evidence that the quercetin spectra is affected at BS concentrations < 2 mM (Fig. 4A–C), which is before any micelles have been detected by pyrene (Fig. 2), strongly suggesting that quercetin is interacting with monomeric BS. This lead to uncertainty in the cmc values which are estimated to be within about 10% for BS micelles and 5% for SDS micelles.

The use of flavonoids to measure cmcs has not been previously reported. It is reassuring that both quercetin and pyrene report similar cmcs and our bile salt data agrees with what we have measured (unpublished results) with another probe, rhodamine 6G, by the method of Carey and Small (1969). Our value of the cmc for SDS is also in the same range as measured by other workers (Baloch et al., 2002, Lin et al., 1996), bearing in mind the effects of temperature and ionic strength on the aggregation (Chaudhuri, Haldar, & Chattopadhyay, 2009).

Our data show that the bile salt mixture used here exhibited a higher cmc than obtained for SDS under the conditions studied. This reflects the greater cooperativity of SDS micelle formation. The data determined by UV–visible absorption spectra of quercetin (Fig. 4) are confirmed by pyrene fluorescence measurements, which are shown in Fig. 2. For BS the rise in fluorescent peak ratio (F_R_) coincides with the increase in slopes of the quercetin λ_max_ against surfactant concentration (Fig. 4). However, there are also some clear differences in the behaviour of quercetin between BS and SDS. For example, at pH 7.15 a small increase in λ_max_ for peaks A and B is observed for BS (Fig. 4A and B), whereas no change in λ_max_ occurs for either peaks for SDS (Fig. 4D and E). However, the partition of HQ into SDS micelles at this pH is shown by the sigmoid increase in *r* value for increasing SDS concentration (Fig. 4F) and peak narrowing of peak B (see Supplementary Material).

Another clear difference in the behaviour between BS and SDS is the magnitude of the response with both pyrene fluorescence and quercetin absorption. The magnitude of the increase in F_R_ of pyrene is greater for BS, with a plateaux value of around 1.25 (Fig.2A), compared to a plateaux value of less than 1.0 for SDS (Fig.2B). This suggests that the environment of the pyrene in the BS micelles is less polar than for SDS. Similarly, the magnitudes of increase in λ_max_ for quercetin is greater in the presence of BS for both peaks A and B (Fig. 4A and B) than they are in the presence of SDS (Fig. 4D and E) at all pH values, again suggesting some difference in the environment of the quercetin between the two surfactant micelles.

### 4.2 How peaks A and B report on quercetin’s different environments

At pH 7.15, 54% of the quercetin molecules will be ionised. Ionised quercetin (Q^−^) will have very little affinity for the negatively charged micelles, so unionised quercetin (HQ) preferentially partitions into the micelles. Depleting the aqueous environment of free HQ will force the equilibrium towards HQ giving a decrease in concentration of Q^−^ and attenuation of the 270 nm peak.

$${\mathrm{HQ}}_{\mathrm{micelle}}⇌\mathrm{HQ}\;⇌\;H^{+}+Q^{-}$$

Further, movement of quercetin into the less polar environment of the micelle leads to an increase in its pK_a1_. Both factors act in the same direction on the acid-base equilibrium to increase the absorbance ratio, *r*, of peak A, as shown in Fig. 4C. Therefore, our hypothesis is that peak A reports on quercetin in the aqueous phase; whereas peak B gives information about unionised quercetin in the micelles.

Consistent with this, although peak A’s wavelength of maximum absorption increases with bile salt concentration, the increase is small compared to that of peak B. This implies free quercetin interacts with monomeric bile salts – an idea that explains why below the cmc, as shown by the increase in pyrene F_R_ (Fig. 2), there is a small increase in wavelengths of maximum absorption as the bile salt concentration increases. That peak B also has a contribution from Q^−^ in the aqueous phase, is shown by the peak width narrowing as the surfactant concentration increases (Fig. 3A). Consequently, lowering the pH to 6.1 or 5.0 increases HQ concentration, and an enhanced slope of λ_max_ versus bile salt concentration for peak A below the cmc is observed, confirming the monomeric surfactants’ interaction with quercetin, HQ. The absence of both the enhancement of *r* for peak A (see Fig. 4C, right hand y axis) and a slight narrowing of peak B with bile salt concentration (Fig. 3A and Fig. S2A and B) substantiate this interpretation. Although reduced pH enhances partition of quercetin into the micelles, it has no significant effect on the cmc as measured by the increases in λ_max_ for peaks A and B (Table 1) and the fluorescent ratio for pyrene (see Fig. 2).

### 4.3 Relative polarity of quercetin binding sites in micelles from solvent studies

Our solvent studies on quercetin spectra, which show a bathochromic shift of λ_max_ for peak B as dielectric constant decreases, are consistent with the binding site for quercetin being more non-polar for BS micelles (see Fig. 5). However, when the polarity reported by quercetin and pyrene in the micelles is expressed in terms of dielectric constant the two probes report different values: quercetin in the range 45–50 for BS and 60–65 for SDS (Fig. 5) and pyrene around 2 for BS’s (Dong and Winnik, 1984, Karpovich and Blanchard, 1995) and >2 to 20 for SDS (Dong and Winnik, 1984, Kalyanasundaram and Thomas, 1977, Karpovich and Blanchard, 1995). This reflects the different nature of the interactions with the environment that are being sensed. Quercetin’s UV–visible absorption spectra show how electronic transitions are affected, whereas pyrene’s fine structure of the fluorescence spectra shows the effect on vibrational modes.

### 4.4 Interaction of pyrene and quercetin within micelles

Pyrene fluorescence in the presence of quercetin shows an increase in F_R_, at higher BS and SDS concentrations (Fig. 2). Both probe molecules interact with a region close to the head group region of the micelles. It has been suggested (Liu & Guo, 2006) that quercetin, when binding to the micelles, may force pyrene to move towards the centre of the micelles, a less polar environment. The relative increase in F_R_ is larger for SDS micelles. Therefore, if this shift in pyrene’s position occurs in both types of micelles, we expect a smaller increase in F_R_ for BS micelles, where there is no sharp distinction between the polar and nonpolar domains (Madenci & Egelhaaf, 2010). For bile salt micelles the polarity decrease is only significant at pH values below the pK_a1_, where high HQ concentrations are present. However, the quenching of pyrene fluorescence by quercetin is also consistent with a direct interaction between HQ and pyrene by, for example, energy transfer or exciplex formation. An increase in F_R_ has been observed in the exciplex between pyrene and a nucleobase in DNA (Yamana et al., 1999). Further work is required to determine the precise mechanism of quenching and the micellar location of pyrene in the presence of quercetin.

### 4.5 Advantage of using the bioactive as a probe in re**lation to understanding bioaccessibility**

In order to be bioavailable, compounds in food must be first made bioaccessible. For lipophilic molecules, bioaccessibility depends on solubilisation in micelles. The advantage of our approach (using quercetin as an intrinsic probe) is that its interaction with micelles can be studied directly without recourse to other methods.

Our observations throw light on why the bioavailability of quercetin is so low. The pH at the site of absorption in the small intestine in the fed state varies between individuals and depends on the nature of the meal. However, as a consensus view, during initial stages of digestion, the pH in the duodenum is around 7, close to the pK_a1_ of quercetin (Minekus et al., 2014). Therefore, only half the quercetin is available for solubilisation in the micelles. As digestion proceeds, due to release of fatty acids the pH may fall to less than 6 (Kalantzi et al., 2006). Then the quercetin becomes increasingly insoluble and its absorption dependent on solubilisation in micelles. As well as simple micelles, mixed micelles provide a solubilisation pathway. These are also negatively charged and therefore a similar dependence of their solubilisation capacity on pH is expected. Nevertheless, to form a complete picture of quercetin’s bioaccessibility our ongoing studies on quercetin in mixed micelles are of fundamental importance. Our methods can be applied to other lipophilic bioactives, which have intrinsic properties that report on their environments.

## 5 Conclusion

Based on its UV–visible properties, we have used quercetin as an intrinsic probe to measure its environment in BS and SDS micelles. We found the cmc is lower for SDS compared to that for BS’s, reflecting a greater cooperativity for SDS micelle formation. Pyrene fluorescence data confirm the cmc values. The relative polarities of the micellar binding site reported by quercetin indicate that SDS has the more polar site compared to BS’s. Under physiological conditions and a BS mixture mimicking the polarity of *in vivo* bile salts in the human duodenum, we show that only unionised quercetin partitions into both BS and SDS micelles. In the presence of micelles, quenching of pyrene fluorescence by quercetin means there is uncertainty about whether pyrene’s location in the micelles is altered by the presence of quercetin. Although quercetin circulating in the blood is in the form of quercetin conjugates, together with methylated and sulphated derivatives, the aglycone is absorbed from the small intestine. Therefore, the results are of relevance to understanding the low *in vivo* bioaccessibility of quercetin.

## Figures and Tables

**Table 1:** Characteristics of micelle formation for bile salt (BS) and sodium dodecyl sulphate (SDS) using quercetin as a probe.

	[Quercetin] (μM)	pH	CMC (mM)	[S]_1/2_ (mM)	λ_max_ (nm)	r_max_	Slope at [S]_1/2_ (r)	Slope at [S]_1/2_ (λ_max_) (nm mM^−1^)
*Peak A*								
BS	45	7.15	1.8 (r)	4.08 (r)		1.02	0.24	
BS	45	7.15	1.8	3.78	257.7			0.72
BS	45	6.1	1.9	3.69	257.7			1.75
BS	22.5	6.1	1.9	3.24	257.7			1.42
BS	22.5	5.0	2.0	3.51	257.7			1.54

SDS	45	7.15	1.0 (r)	3.43 (r)	–	1.27	0.11	
SDS	45	6.1	1.1	2.56	255.6			1.09

*Peak B*								
BS	45	7.15	1.8	2.78	377.9			3.11
BS	45	6.1	1.7	3.11	376.3			4.21
BS	22.5	6.1	1.7	3.17	376.2			4.34
BS	22.5	5.0	1.8	2.27	375.8			3.76
SDS	45	6.1	1.0	1.97	373.2			8.65

CMC starting concentration for micelle formation; [S]_1/2_, inflection/ midpoint of sigmoid curve for micelle formation; λ_max_, wavelength of maximum absorption; r_max_, the maximum ratio of A_max(254–257)_/A_270_; slope at [S]_1/2_, fitting parameter for calculation of [S]_1/2_ using r and λ_max_; results are the mean of 2–3 measurements with SD for the values within 5% (SDS) to 12% (BS) for cmc’s, within 2% (SDS) to 10% (BS) for [S]_1/2_, within ±0.5 nm for λ, within 3% for r_max_ and within 10% for slopes
