# Supplementary material for: Quercetin solubilisation in bile salts: A comparison with sodium dodecyl sulphate
Source: Food Chem. 2016 Nov 15;211:356–64. doi: 10.1016/j.foodchem.2016.05.034 (PMC4911888; doi:10.1016/j.foodchem.2016.05.034)
Supplement: Supplementary data [file mmc1.docx]

**Supplementary data**

Fig. 1a shows the effect of pH on the wavelength of maximum absorption, λ_max_, for quercetin’s peak B. Above pH 7.5, λ_max_ is influenced by the second dissociation of quercetin to Q^2-^. λ_max_ for unionised quercetin, HQ, is 366.7 + 0.4 nm; λ_max_ for Q^-^ was estimated to be 382 nm. Assuming linearity the concentrations of HQ and Q- are proportional to (382 - λ_max_) and (λ_max_ -366.7), respectively. These differences in wavelength, Δλ, are plotted in fig. 1b. When the pH is equal to quercetin’s pKa, the concentrations of HQ and Q- are equal and Δλ for the curves intersect.

**Fig. 1a.** The wavelength of maximum absorption, λ_max_, for peak B as a function of pH.

(λ_max_-366.7)

(382 – λ_max_)

**Fig. 1b.** The pH dependence of changes in wavelength of maximum absorption for unionised, HQ, and ionised, Q^-^, quercetin. (382 - λ_max_ ) is proportional to the concentration of HQ and (λ_max_ - 366.7) is proportional to the concentration of Q^-^.

Figs 2a and 2b show UV-visible spectra of quercetin in bile salt micelles at pH 6.1 and 5.0, respectively.

**[BS] mM**

**pH 6.1**

**Fig. 2a**

**[BS]** **mM**

**pH 5.0**

**Fig. 2b**
